# Assessing the predictability of self-harm in a high-risk adult prisoner population: a prospective cohort study

**DOI:** 10.1186/s40352-018-0076-3

**Published:** 2018-09-21

**Authors:** Mike C. Horton, Wendy Dyer, Alan Tennant, Nat M. J. Wright

**Affiliations:** 1 Section of Rehabilitation Medicine, Leeds Institute of Rheumatic and Musculoskeletal Medicine, Faculty of Medicine and Health, University of Leeds, D Floor, Martin Wing, LGI, LS1 3EX, Leeds, UK; 2 School of Arts and Social Sciences, Northumbria University, Lipman Building Room 216, Newcastle Upon Tyne, NE1 8ST UK; 3 Swiss Paraplegic Research, Guido A. Zäch-Strasse 4, 6207 Nottwil, Switzerland; 4 Clinical Research Director Transform Research Alliance, Visiting Associate Professor Leeds University, Spectrum Community Health CIC, One Navigation Walk, Hebble Wharf, Wakefield, WF1 5RH UK

**Keywords:** Self-harm, Prison, Assessment, Prediction, Screening, Clinical decision aid, Risk indicators, Protective indicators

## Abstract

**Background:**

Prisoners are at increased risk of self-harm and when either intent is expressed, or an act of self-harm carried out, prisoners in the UK are subject to self-harm/suicide monitoring (referred to as “open ACCT” monitoring). However, there is a paucity of validated instruments to identify risk of self-harm in prisoner populations. In response to the need to support prison staff to determine who is at increased risk of self-harm or repeat self-harm, the aim of this study was to determine whether any pre-existing, standardised instruments could usefully identify future self-harm events in prisoners undergoing ACCT monitoring.

**Methods:**

A multi-stage prospective cohort study was conducted, where the Prison Screening Questionnaire (PriSnQuest), a modified Borderline Symptom List-23 (BSL-23), Self-Harm Inventory (SHI), Patient Health Questionnaire-9 (PHQ-9) and Clinical Outcomes in Routine Evaluation – Outcome Measure (CORE-OM) instruments were administered to prisoners aged 18 and above, who were judged to be at an increased risk of self-harm (on open ACCT monitoring) during the recruitment phase. A 6-month follow-up determined self-harm occurrence since baseline, and Area-Under-the-Curve (AUC) analysis examined the ability of the instruments to predict future self-harm.

**Results:**

Prison records established that 29.1% self-harmed during the follow up period, involving a total of 423 self-harm events reported from 126 individuals, followed up for 66,789 prisoner days (median 167 days; IQR 71–207.5 days). This translated to an ‘event incidence’ of 6.33 per 1000 prisoner days of those who had been placed upon an ACCT, or ‘prisoner incidence’ of 1.89 per 1000 days, with considerable variation for both gender and participating prisons. None of the summary scores derived from the selected instruments showed a meaningful ability to predict self-harm, however, exploratory logistic regression analysis of individual background and instrument items revealed gender-specific item sets which were statistically significant in predicting future self-harm.

**Conclusions:**

Prospective self-harm was not predicted by any of the pre-existing instruments that were under consideration. Exploratory logistic regression analysis did reveal gender-specific item sets, producing predictive algorithms which were statistically significant in predicting future self-harm; however, the operational functionality of these item sets may be limited.

## Background

**Fig. 1:**
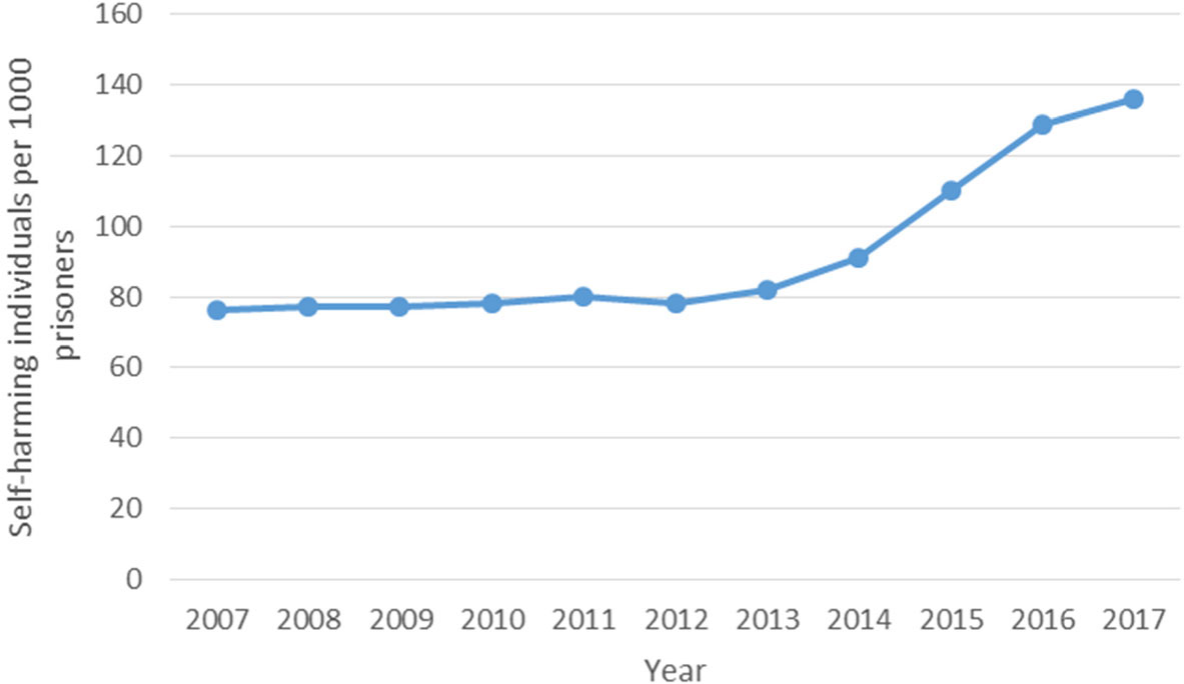
Rate of self-harming individuals per 1000 prisoners, from 2007 to 2017

Prisoners have increased risk of self-harm (Fazel et al. 2011; Hawton et al. 2014; Ministry of Justice 2018a) which is defined by NICE as any intentional self-poisoning or self-injury, irrespective of the degree of suicidal intent or underlying motive (NICE 2011). This corresponds to the definition of self-harm used within prison custody, where it is defined as, *“any act where a prisoner deliberately harms themselves irrespective of the method, intent or severity of any injury”* (Ministry of Justice 2018b). Although active definitions of self-harm vary among studies and reports, international statistics highlight a prisoner self-harm rate of 100 per 100,000 prisoners, which is significantly higher than the rate of 21 per 100,000 found in the general community (Fazel et al. 2011). Current UK prison figures suggest a much higher rate than this, with 136 self-harming individuals per 1000 prisoners in 2017 (Ministry of Justice 2018a), with an increasing trend (See Fig. 1). This overall prison rate increase is fully attributable to a rate increase among males (Ministry of Justice 2018a). Over the last 10 years (2007–2017) the amount of self-harm incidents in male prisons has trebled, and the rate of self-harming individuals per 1000 male prisoners has doubled (from 63 to 128) (Ministry of Justice 2018a). Although self-harm rates among female prisoners have remained largely stable over the last 10 years, they continue to account for a disproportionate amount of self-harm in prison custody – accounting for around 5% of the prison population but 20% of self-harm incidents. However, not all prisoners experience the same level of risk, and it is known that there are a small number of prisoners who are responsible for a large number of self-harm events (Hawton et al. 2014).

In order to target this issue, self-harm was included in the NHS England (2013) service specification for public health services for people in prison (NHS 2013), and the Public Health Outcomes Framework 2013 to 2016 (Department of Health 2013) as part of the ‘Health Improvement’ domain. However, reports by the Prison and Probation Ombudsman (Prison and Probation Ombudsman 2014a, 2014b, 2014c) continue to raise concerns, and although the Public Health England (2015) ‘Health and Justice 2014’ report (Public Health England 2015) acknowledged this rise in prisoner self-harm, there has actually been a surge in prisoner self-harm since its publication (see Fig. 1).

Although it seems to be escalating, the issue of self-harm in prisons is not a new problem. In recognition of the issue, in 2005 the Prison Service piloted a care-planning system called ACCT (Assessment, Care in Custody, and Teamwork) (HM Prison Service and Safer custody group 2005) to improve the care for prisoners at risk of suicide or self-harm, and this was implemented nationally in 2007. Complete details of the process are available elsewhere (Humber et al. 2011), but the ACCT document is designed to ensure prison staff keep a concise record of the prisoner’s care, needs and problems. An important point to mention is that a prisoner only needs to be considered as ‘at risk’ for an ACCT to be opened, and the reasons for this are variable. Although an ACCT would be opened if a prisoner carried out a self-harm incident, many ACCTs are opened without any incidence of self-harm. The initial ACCT assessment effectively establishes a care pathway system for those deemed to be at risk. However, it does not incorporate a standardised diagnostic test to estimate the risk of future self-harm.

A time of particular vulnerability for prisoners is upon reception into prison, where it has been identified that a third of all prison suicides take place in the first 7 days (Shaw et al. 2004). Due to the increased vulnerability of prisoners during the reception period, all new prisoners are screened using a standardised prison questionnaire which was designed to screen for physical and mental health problems (Gavin et al. 2003). Although this screening tool is not intended to predict the risk of self-harm or suicide, it does allow for the broad identification of high-risk problems such as self-harm or suicide risk, which may warrant further assessment. If a risk of self-harm or suicide is deemed to be present, this would also trigger the opening of an ACCT document (Humber et al. 2011). In 2017, 8 % of the total self-harm incidents occurred within the first 7 days of reception into prison (Ministry of Justice 2018a), but this value could potentially be much higher if the reception screening process were not in place.

There is some evidence to suggest that the reception screening tool can help identify true cases of psychiatric illness upon entry into prison (Gavin et al. 2003). This early indication of mental and physical health problems is beneficial to prison staff in terms of prisoner management, but the key issue remains as to whether individuals specifically at risk of self-harm or suicide can be identified at reception into prison (Hawton et al. 2014). Early recognition of this risk could lead to increased staff awareness and the initiation of appropriate preventative measures being put in place; therefore potentially lowering the rate of self-harm and reducing the demand on the prison healthcare system (Lohner and Konrad 2007).

One way to approach the development of a screening process specific to self-harm, would be to assess the associated risk factors for self-harm. However, it is necessary for these risk factors to be statistically obtained, as clinical intuition is a notoriously error-prone practice of risk assessment (Haycock 1989; Lohner and Konrad 2007). Risk factor studies are indispensable to broaden our knowledge of self-harm (Lohner and Konrad 2007), and they have been used to generate self-harm screening algorithms specifically for prison populations (Blaauw et al. 2005; Lanes 2009), although these have not been tested prospectively. Also, with regard to the majority of the risk factors that have been identified specifically to self-harm in prisons, a major problem is that there is also conflicting evidence to disregard these same risk factors (Lohner and Konrad 2007). This is possibly because a lot of the factors that have been identified as associated with self-harm are non-specific, and are therefore of limited value (Hawton et al. 2014).

The evidence to support the routine use of any screening instrument for self-harm in incarcerated adult populations is limited, and the transferability of any existing self-harm screening instruments is problematic due to the unique environment in which prisoners are accommodated (Perry et al. 2010). A review article identified four screening instruments across five studies that have been used to assess for the risk of suicide and self-harm in incarcerated adults, although three of these instruments were specifically aimed at screening for suicide (or suicide risk) rather than self-harm (or risk of self-harm), and two of the studies used retrospective methodology which may result in non-comparable information between study participants (Perry et al. 2010). Additional limited evidence suggests that the Beck Depression Inventory (Beck et al. 1961) may be predictive of self-harm behaviour among female prisoners (Perry and Gilbody 2009), and that the Beck Hopelessness Scale (Beck et al. 1974) may be predictive of self-harm among incarcerated adults with mental disorders (Gray et al. 2003), but not among female prisoners (Perry and Gilbody 2009). One scale, Suicide Concerns for Offenders in Prison Environment (SCOPE) (Perry and Olason 2009) has been specifically developed to assess vulnerability to risk of suicide and non-fatal self-harm behaviour in young incarcerated adults. However, again, this has not been tested with regard to implementation for routine prison use or as part of the ACCT process, and although it does demonstrate some evidence for its prospective predictive validity, this was only demonstrated in a female cohort (Perry and Gilbody 2009).

Self-harm remains a significant, growing problem in prisons, and the identification of those most at risk would help towards the introduction of timely coping strategies which could be key for the successful management of self-harm within a prison setting, as self-harm is associated with a disproportionate utilisation of health resources (Smith and Kaminski 2010). If a useful screening instrument could be identified, this could provide an opportunity for early recognition of risk (Lohner and Konrad 2007; Morgan and Hawton 2004). If this were done in a standardised way this may also provide legal protection (Lohner and Konrad 2007; O'Leary 1989), as it has been identified that prison professionals have often been unfairly criticised for not identifying this risk, particularly when a prisoner self-harms following closure of an ACCT (Wright et al. 2012).

In response to the need to support prison staff to determine who is at increased risk of self-harm or repeat self-harm, the aim of this study was to determine whether any pre-existing, standardised instruments could usefully identify future self-harm events in prisoners undergoing ACCT monitoring.

## Methods

### Study design

A multi-stage prospective cohort study was undertaken. This included: a scoping study to select the instruments to be tested; a pilot study to refine the research protocol, the choice of instruments, and the operational issues around decision making in a prison environment; and a cohort study where instruments were administered at baseline, with a 6 month follow-up to determine self-harm occurrence since baseline. Area-Under-the-Curve (AUC) analysis examined the ability of instruments to predict future self-harm.

### Procedures

Following ethical and governance approval, participants were recruited from three remand (i.e. not training/resettlement) adult prisons in Northern England: one female closed prison, and two male Category B (closed, for those who do not require maximum security, but for whom escape still needs to be made very difficult) prisons. Eligibility criteria included prisoners aged 18 and above, who had an ACCT opened during the recruitment phases. The ACCT population were targeted due to the increased self-harm event rate compared to the overall prison population (a brief audit of the three prisons suggested that on average approximately 20% of inmates are assigned an ACCT in any given year, but the incidence of self-harm following an ACCT was not known).

The scoping exercise systematically identified existing potential instruments through searching the SCOPUS database, grey literature, and internet. The search yielded 955 journal article records which revealed 130 potential instruments regarding self-harm or suicide. Selection of potential instruments was by a group of professionals with expertise in delivery of prison health care, psychometrics or as a service user representative. To be considered for the study, each potential instrument had to satisfy certain practical criteria, including: the instrument must be able to be administered by generic primary care/prison/research staff that may not have had mental health or clinical training, or any specialist training specific to the instrument; the instrument must be able to be administered orally by staff rather than self-administered (to account for issues regarding literacy); the instrument must not be specifically designed for administration *following* a self-harm event (people at risk may or may not have actually carried out a self-harm incident); the instrument must be comprised of closed questions with a discrete response format to allow for objectively measured responses and consistency among respondents; the instrument must be brief, in line with the circumstances in which it would be administered in a prison environment; any instruments containing more than 50 individual questions were excluded as inappropriate; the instrument must be available for use within the study.

Eight instruments were piloted to determine operational aspects of the study, test follow-up processes and provide an estimate of the incidence of self-harm during follow-up for cohort study power calculations. A cognitive debrief also followed each prisoner interview, to collect feedback on the acceptability of the administered instruments.

Following the pilot study, five instruments were selected based on pilot participant feedback and the views of the expert panel. The final set of five instruments reflected the range of potential pathologies which could contribute to self-harm and included: the Prison Screening Questionnaire (PriSnQuest) (Shaw et al. 2003); a modified version of the Borderline Symptom List − 23 (BSL-23) (Bohus et al. 2009), (amended to measure frequency rather than intensity of symptoms – referred to as BSL-23-F); the Self Harm Inventory (SHI) (Sansone et al. 1998); the Patient Health Questionnaire (PHQ-9) (Kroenke et al. 2001); and the Clinical Outcomes in Routine Evaluation Outcome Measure (CORE-OM) (Evans et al. 2000). The instruments that were eliminated were the Beck Hopelessness Scale (BHS) (Beck et al. 1974), the Suicide Concerns for Offenders in Prison Environment (SCOPE) (Perry and Olason 2009), and the Depression, Anxiety & Stress Scale (DASS-21) (Lovibond and Lovibond 1995). The BHS was removed on the basis of prisoner respondent feedback, which indicated that some of the questions were found to be confusing. It was also thought that a lot of the questions could be taken out of context when applied within a prison setting. The SCOPE was removed due to a confusing, inconsistent response structure, along with questions that were not applicable to a range of respondents. There were no specific issues found with the DASS-21, but it was eliminated in favour of the PHQ-9 and the CORE-OM, both of which covered similar content to the DASS-21, the former already widely used within UK Primary health care.

Findings from the pilot study also informed the decisions to increase the time period between opening ACCT and recruiting into the study from the initial target of 48 h to 2 weeks, as 48 h proved to be logistically impractical, and a 3 week time frame still falls within the stated range of each included instrument. Additionally, the follow-up period was reduced from 9 months to 6 months (of 75 people recruited to the pilot study, 40% self-harmed during follow-up, and of these 96.7% did so within 6 months). The pilot study also informed that a sample size of 359–475 would give 80%–90% power for the area under the curve (AUC) analysis (assuming a conservative rate of 30% for self-harm, and a 6 month follow up period with a 20% loss to follow-up rate).

At baseline, the study researchers administered the five instruments in their complete form, within a standardised questionnaire format which also covered sociodemographic and sentencing information. Participant feedback from the pilot study suggested that this was not a burdensome process, despite the length of the questionnaire. The active follow-up period was variable, with this being either up to the point of release from prison, or 6 months after baseline where the prisoner is still within the prison system. All follow up data pertaining to self-harm was retrieved from prison safer custody records.

### Statistical analysis

Each of the five instruments was analysed for their predictive capabilities regarding future self-harm events using AUC analysis. All initial statistical analyses were carried out using SPSS version 21 (IBM SPSS Statistics for Windows 2012).

## Results

590 prisoners were eligible for inclusion, of which 452 (76.6%) consented, although two prisoners subsequently withdrew consent. Recruitment rate was similar across prisons, ranging from 70.7%–79.0%. The mean age was 31.2 years, and 26% were female. Prisoner demographics can be found in Table 1.

**Table 1:** Demographic and sentence characteristics of participants recruited - significance across prisons

Characteristic	Prison A	Prison B (female)	Prison C	Total	Significance^a^	*N*
Mean Age (Years)	31.2	29.6	32.0	31.2	0.102	450
Age leaving FT education	15.3	15.5	15.3	15.3	0.896	440
% without any educational qualifications	26.7	36.8	55.3	43.8	< 0.001	447
Have Children (%)	51.4	44.3	51.1	49.4	0.447	449
Received visit in last 7 days (%)	15.2	14.8	13.6	14.3	0.858	448
% on remand	56.2	22.6	52.2	45.6	< 0.001	245
Of those sentenced						
- Tariff in months	53.8	44.6	32.1	41.0	0.394	225
- Served	9.8	17.2	14.8	14.7	0.388	239
*N*	105	115	230	450		

^a^ F-test for continuous variables; Chi-Square for proportions

Just over one third of ACCTs had been initiated because of a known self-harm event. Seventeen (3.8%) participants were lost to follow-up and 29.1% self-harmed during the follow up period (the most common self-harm behaviour during follow-up was cutting). Overall, 46.7% of those entered into the study self-harmed, either at the time of their Index ACCT, or in the follow-up period. During the follow up period (Table 2) a total of 423 self-harm events were reported from 126 individuals, followed up for 66,789 prisoner days (median 167 days; IQR 71–207.5 days). This translated to an ‘event incidence’ of 6.33 per 1000 prisoner days *of those who had been placed upon an ACCT*, or ‘prisoner incidence’ of 1.89 per 1000 days. However, this is only the average from the current study, it varies considerably by gender (see Table 2), and also between prisons.

**Table 2:** Incidents of self-harm during follow-up – by prison and gender

	Prison A	Prison B (Female)	Prison C	Total	Male Prisons
*N*	105	115	230	450	335
*N* with valid follow up	102	111	220	433	322
Total number of self-harm events reported during follow-up	50	207	166	423	216
Total number of prisoner follow-up days	13,470	13,074	40,245	66,789	53,715
Event Incidence per 1000 prisoner-days	3.71	15.83	4.12	6.33	4.02
Total number of people with self-harm events reported during follow-up	17 (16.7%)	37 (33.3%)	72 (32.7%)	126 (29.1%)	89 (27.6%)
Person self-harm Incidence per 1000 prisoner-days	1.26	2.83	1.79	1.89	1.66
self-harm event – person ratio	2.94	5.59	2.31	3.36	2.43

All instruments showed some support for unidimensionality, and four-out-of-five showed scaling criteria consistent with ordinal scaling, so verifying the validity of cut points (the exception being the CORE-OM) (Horton et al. 2014). However, none of the summary scores from the instruments displayed a meaningful AUC value (Horton et al. 2014). Due to gender differences in the patterns of self-harm and gender biases within some of the instruments, this analysis was repeated for males and females, which also failed to display any meaningful AUC value (Horton et al. 2014). The highest AUC value reported was 0.671 for the SHI in the female analysis. Although this was reported as statistically significant, the AUC predictive value is still classified as ‘poor’ (Metz 1978). Additionally, Rasch (Rasch 1960) analytic techniques were used to refine each of the pre-existing instruments in terms of their measurement properties, but this did nothing to improve any of the AUC predictive values (Horton et al. 2014).

### Exploratory analysis of predictive items

Although none of the summary scores derived from the selected instruments showed a meaningful ability to predict self-harm, these instruments do contain a range of individual items that may be usefully predictive risk indicators. The 105 items from the candidate instruments, together with other socio-demographic and sentencing criteria, were therefore investigated in an exploratory manner, in order to assess their potential as individual predictors of risk.

This item set was initially reduced to contain only those items which had potentially indicated risk of self-harm (i.e. those items that were individually associated with future self-harm at *p* = 0.10 as indicated by crosstab chi-square tests). In order to present an example of the type of items remaining in this set, those individual items that were statistically significantly associated with future self-harm at *p* = 0.05 are included in Table 3. This analysis was undertaken separately for the male and female samples.

**Table 3:** Items and other indicators associated (*p* < 0.05) with future self-harm by gender

Variable (Odds Ratios refer to affirmation of variable)	*p*-value	OR	CI	Sensitivity	Specificity	PPV	NPV
Male Risk Factors							
Prisoner has no qualifications (no qualifications = 1)	0.001	2.335	1.409–3.872	62.10%	58.80%	36.00%	80.60%
BSL1. In the last week it was hard for me to concentrate	0.017	> 100	not calculated	100%	6.50%	27.80%	100%
BSL S8. During the last week I had uncontrollable sexual encounters of which I was later ashamed or which made me angry	0.028	8.475	0.869–82.65	3.60%	99.60%	75%	73.90%
Ever Self-Harmed in prison	0	3.423	1.967–5.958	75.90%	52.10%	37.10%	85.30%
Index ACCT due to Self-Harm?	0	3.42	1.986–6.836	71.40%	59.60%	46.70%	80.80%
SHI2. Have you ever cut yourself on purpose?	0	3.075	1.604–5.894	84.10%	36.70%	32.20%	86.60%
PQuest2. In the past year have you been taking longer over the things you do?	0.024	2	1.086–3.685	80.70%	32.30%	29.90%	82.40%
Ever received medication for mental health problems	0.023	1.981	1.091–3.596	80.50%	32.50%	30.70%	81.70%
SHI19. Have you ever exercised an injury on purpose?	0.045	1.858	1.007–3.427	25.30%	84.60%	37.50%	75.60%
PQuest1. In the past year have you previously seen a psychiatrist?	0.018	1.821	1.105–3.001	54.70%	60.20%	33.80%	78.10%
Acquisitive Crime (Burglary, Robbery, Theft)	0.043	1.712	1.015–2.89	38.80%	73.00%	34.40%	76.60%
Male Protective Factors							
Age left full time education (16+ = 1)	0.034	0.578	0.348–0.962	37.60%	48.90%	21.30%	68.10%
SHI6. Have you ever Abused alcohol?	0.028	0.559	0.332–0.941	57.80%	28.90%	22.90%	65.30%
Dependent on alcohol	0.013	0.497	0.284–0.867	24.10%	60.90%	18.80%	68.30%
CORE19. Over the last week I have felt warmth or affection for someone (with scoring reversed) 1 = Less than all the time	0.003	0.476	0.288–0.786	49.40%	32.80%	21.60%	63.30%
Female Risk Factors							
Life or indefinite sentence	0	8.4	2.479–28.46	32.40%	94.60%	75%	73.70%
SHI2. Have you ever cut yourself on purpose?	0.01	4.795	1.331–17.269	91.90%	29.70%	39.60%	88%
PHQ-9-7. Over the last 2 weeks - Trouble concentrating on things, such as reading the newspaper or watching television	0.04	4.449	0.96–20.619	94.60%	20.30%	37.20%	88.20%
PQuest8. In the past year have you recently heard voices saying a few words or sentences when there was no one around to account for this?	0.001	4.19	1.768–9.928	73%	60.80%	48.20%	81.80%
CORE 25. Over the last week I have felt criticised by other people	0.003	3.544	1.501–8.366	73%	56.80%	45.80%	80.80%
SHI3. Have you ever Burned yourself on purpose?	0.011	3.145	1.269–7.793	37.80%	83.80%	53.80%	72.90%
Ever Self-Harmed in prison	0.05	3.056	0.96–9.723	89.20%	27%	37.90%	83.30%
Index ACCT due to Self-Harm?	0.017	2.9	1.189–7.084	63.30%	62.70%	43.20%	79.20%
SHI8. Have you ever Scratched yourself on purpose?	0.015	2.708	1.203–6.096	59.50%	64.90%	45.80%	76.20%
SHI10.Have you ever made medical situations worse on purpose (e.g. skipped medication)?	0.017	2.664	1.174–6.044	51.40%	71.60%	47.50%	74.60%
BSL S4. During the last week I had episodes of binge eating	0.031	2.609	1.079–6.309	37.80%	81.10%	50%	72.30%
SHI21. Have you ever starved yourself to hurt yourself?	0.022	2.588	1.132–5.918	67.60%	55.40%	43.10%	77.40%
SHI9. Have you ever prevented wounds from healing?	0.032	2.41	1.071–5.419	62.20%	59.50%	43.40%	75.90%
BSL 15. Over the last week I suffered from voices and noises from inside or outside my head	0.032	2.41	1.071–5.419	62.20%	59.50%	43.40%	75.90%
Female Protective Factor							
First time on an ACCT?	0	0.224	0.096–0.523	36.10%	28.40%	19.70%	47.70%

To account for the small number of self-harm cases, all items with multi-category response options were dichotomised into categories that represented a ‘complete absence’ and ‘some presence’ of either a sign or symptom.

Following this initial assessment, the exploratory analysis was extended in order to investigate whether a set of items could be considered together to produce a predictive algorithm. Again, this was undertaken separately for males and females. All pool items which were individually significant at *p* = 0.10 were entered into a backwards stepwise binary logistic regression, under a likelihood-ratio removal process (p removal 0.1) (Field 2005).

Following the initial analysis run, a composite item of ‘Prison self-harm history’ was created from three individual items: ‘Have you ever self-harmed in prison?’, ‘Was the prisoner’s index ACCT due to self-harm?’, and item 1 of the BSL supplement ‘During the last week I hurt myself by cutting, burning, strangling, head banging etc.’. This grouped the prisoners into three categories: those that had never self-harmed in prison; those that had self-harmed in prison, but not recently (not within the previous 2 weeks); and those that had self-harmed in prison recently (within the previous 2 weeks). The composite item was significantly predictive for the male sample, so it was used instead of the constituent items. It was not significantly predictive for the female sample, so the individual items were retained.

Additionally at this point, the male sample statistical analysis software was switched from SPSS to STATA 14 (StataCorp 2015) as STATA offered the opportunity to apply a Firth adjustment (Firth 1993) following the discovery of complete separation within the data set, which can occur when the (self-harm) event numbers are limited. Where complete separation occurs within the data, the maximum likelihood values of the logistic regression cannot be estimated, and the Firth adjustment allows for the convergence of finite estimates, therefore reducing the bias within the analysis (Heinze and Schemper 2002).

The final models contained 11 independent variables for males (Table 4) and seven independent variables for females (Table 5). Both models were statistically significant, (male model: χ2 (df 12, *N* = 301) = 47.57, *p* < 0.001; and female model: χ2 (df 7, *N* = 94) = 53.46, *p* < 0.001) indicating that the models were able to distinguish between prisoners who went on to carry out a self-harm event in the follow-up, and those who did not. Seven of the 11 independent variables in the male model, and five of the seven independent variables in the female model made a unique statistically significant contribution to the final models.

**Table 4:** Logistic regression predicting likelihood of self-harm during follow-up for males

Variable	B	S.E.	z	Sig.	95% C.I.	
					Lower	Upper
Do you have any qualifications? (yes = 0, no = 1)	1.122977	0.31065	3.61	0	0.514114	1.731839
Have you accessed healthcare during this prison stay?	−1.14773	0.442399	−2.59	0.009	−2.01481	−0.28064
In the past year, have you previously seen a psychiatrist?	0.660485	0.308926	2.14	0.033	0.055001	1.26597
Have you ever cut yourself on purpose?	0.785021	0.396684	1.98	0.048	0.007535	1.562508
Have you ever abused alcohol?	−1.06009	0.331151	−3.2	0.001	−1.70914	−0.41105
Have you ever driven recklessly on purpose?	−0.6994	0.32734	−2.14	0.033	−1.34098	−0.05783
Have you ever intentionally exercised an injury to hurt yourself?	0.670756	0.370553	1.81	0.07	−0.05551	1.397025
In the last week have you felt warmth or affection for someone?	−0.532	0.304642	−1.75	0.081	−1.12909	0.065083
In the last week, have you thought that you are to blame for your problems and difficulties	1.029772	0.58036	1.77	0.076	−0.10771	2.167257
In the last week, has it been hard for you to concentrate?	1.998831	1.518229	2.84^a^	0.092^b^	−0.97684	4.974505
Prisoners self-harm history in prison: (‘no prison self-harm history’ is reference category)			11.36^a^	0.003^c^		
self-harmed, but not recently	0.922775	0.557201	1.66	0.098	−0.16932	2.014869
self-harmed recently	1.526448	0.468689	3.26	0.001	0.607833	2.445062
Constant	−4.36395	1.648187	−2.65	0.008	−7.59434	−1.13357

^a^Chi-square value ^b^Firth-adjusted p-value ^c^Overall significance of categorical item

**Table 5:** Logistic regression predicting likelihood of self-harm during follow-up for females

Variable	B	S.E.	Wald	df	Sig.	Odds Ratio	95% C.I. for Odds Ratio	
							Lower	Upper
Life or indeterminate sentence?	2.016	1.143	3.112	1	.078	7.506	.799	70.488
Has prisoner had ANY sort of correspondence during stay (yes or no)?	3.698	1.985	3.471	1	.062	40.351	.825	1973.135
Have you ever seen a psychiatrist outside prison?	1.453	.739	3.867	1	.049	4.274	1.005	18.183
Is this the first time in this sentence that you have been put on an ACCT?	−2.027	.762	7.086	1	.008	.132	.030	.586
Have you ever intentionally scratched yourself on purpose?	2.362	.740	10.200	1	.001	10.617	2.491	45.252
During the last week I had episodes of binge eating.	2.714	.867	9.806	1	.002	15.096	2.761	82.544
During the last week I took medication that had not been prescribed or if had been prescribed, I took more than the prescribed dose.	2.213	.878	6.349	1	.012	9.139	1.635	51.093
Constant	−7.022	2.400	8.563	1	.003	.001		

**Fig. 2:**
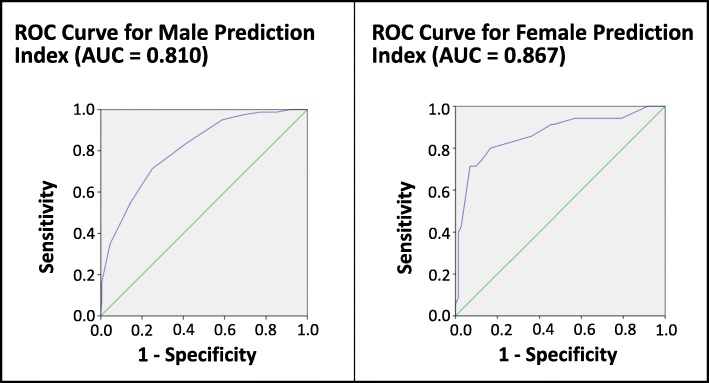
ROC curves for Male and Female predictive risk models

For each prisoner on an ACCT, a risk score can be calculated by multiplying each variable with the regression coefficient of the prediction model. To create a more easily applicable prediction rule, regression coefficients were rounded to half points and then doubled to form simple summative indices of complete numbers. This was done separately for males and females. The receiver operating characteristic (ROC) curves for these prediction models are displayed as Fig. 2. When maximising the Kappa value in the agreement between the prediction model and the outcome of self-harm, corresponding AUC values are 0.81 for males and 0.867 for females. The properties of the gender-specific predictive models are summarised in Table 6.

**Table 6:** Properties of the gender-specific predictive models

Predictive Algorithm	AUC	Sensitivity	Specificity	PPV	NPV	Correctly Classified
Male	0.81	55%	85.5%	57.9%	84%	77.4%
Female	0.867	71.4%	93.1%	83.3%	87%	86%

For these values that are presented, it should be noted that specificity and sensitivity are properties of the instrument, whereas positive-predictive value (PPV) and negative-predictive value (NPV) differ by the self-harm prevalence rate within a given population. As the self-harm rate varied by prison, the PPV and NPV will therefore differ across institutions, although this will only apply to the male institutions as the female institution was considered separately.

For the sensitivity and specificity values obtained within the male prisons, where the self-harm prevalence rate is lower (i.e. Prison A), the PPV will also be lower, but the NPV will be higher. This means that there will be a higher proportion of false positive results of the screening test, but a lower proportion of false negatives. Where the self-harm prevalence rate is higher (i.e. Prison C), the PPV will also be higher, but the NPV will be lower. This means that there will be a lower proportion of false positive results of the screening test, but a higher proportion of false negatives.

By examining crosstabs of different cut points relative to the sensitivity and specificity achieved, it is possible to create a low-medium-high risk classification for the risk of self-harm. A ‘low’ risk classification seeks to maximise the sensitivity of the prediction model, meaning that among those that do self-harm, their identification is maximised. This provides a low cut-point (for males < 2, for females < 3), above which true positive identification is maximised. This cannot be used as single cut point as it also maximises the amount of false positives, but it is useful as it minimises the false negatives identified (i.e. anyone below the cut point value is highly unlikely to self-harm). A ‘high’ risk classification seeks to maximise the specificity of the prediction model, meaning that among those that do not self-harm, their identification is maximised. This provides a high cut-point (for males 10+, for females 16+), below which true negative identification is maximised. This cannot be used as single cut point as it also maximises the amount of false negatives, but it is useful as it minimises the false positives identified (i.e. anyone above the cut point value is highly likely to self-harm). When all individuals are classified (post-hoc) within these risk categories, both genders have a minimal level of self-harm among those categorised as low risk (0% self-harm reported), and those classified as high risk subsequently self-harmed in 73.7% of the male cases, and 88.2 of the female cases. This categorisation by level of risk could contribute to identifying appropriate care pathways and, given the strength of the negative tests, may facilitate sign-off from the ACCT. It is plausible that the respective gender-specific item sets, which resulted from the logistic regression, could form single page clinical decision aids which could be administered by any prison staff within a few minutes.

## Discussion

The basic self-harm incidence during the six-month follow-up was 29.1%, although this value was variable across prison and gender. The overall incidence rate recorded for males was 27.6%, which is more than double the self-harm incidence rate of 12.8% recorded among the general male prison population in 2017 (Ministry of Justice 2018a). This difference in rates would probably be expected, given the difference of study populations. For females, the overall incidence rate recorded during follow-up was 33.3%, which is not markedly higher than the self-harm incidence rate of 30% recorded among the general female prison population in 2017 (Ministry of Justice 2018a), suggesting that the ACCT population in female prisons may appear to be quite similar to the more general female prison population in terms of self-harm activity. It is speculated that, when compared to males, this closer similarity of female self-harm rates is due to a higher proportion of the total female prison population also falling into the corresponding ACCT population.

The primary aim of the study was to determine whether any pre-existing instruments could predict self-harm among an ACCT population. The AUC analysis that was carried out on the candidate instruments determined that none of these performed the task adequately enough to be considered a useful aid for prison staff to utilise as part of a standardised ACCT process. This finding has also been the case when using standardised measures to predict suicide following self-harm, where it has been warned that the use of these standardised scales, or an over-reliance on the identification of risk factors in clinical practice, may provide false reassurance that could be potentially dangerous (Chan et al. 2016).

With regard to the results obtained, it is acknowledged that a potential ‘risk paradox’ issue may also need to be considered: When an individual is identified as being at risk by one (or more) of the instruments that are being assessed, if risk is detected (especially in the case of self-harm risk), then generally something will be done in order to alleviate this risk in the individual. In turn, any element of risk reduction for a given individual may also reduce the probability of the final outcome occurring in the population of interest, thus interfering with any attempts to establish the predictive validity of the instruments that are being assessed. Although this issue may be present, in this instance it is unlikely to have had a major impact on the results as all study participants are from the prison-ACCT population, and are therefore already classified as being at an increased risk of self-harm.

A further potential limitation lies with the self-harm outcome data coming exclusively from prison records. This will likely lead to an under-ascertainment of self-harm events, as some self-harm remains self-managed and unreported. This has been previously observed (Borschmann et al. 2017), and it has been identified that self-harm may be more difficult than other clinical phenomena to measure accurately through medical records (Fliege et al. 2006). Although none of the pre-existing standardised instruments predicted the risk of self-harm in the ACCT population, an exploratory logistic regression revealed a set of items that may be useful when aggregated into a predictive algorithm, which could be used as a clinical decision aid to indicate risk of future self-harm. This risk factor approach has often been used to incorporate individual risk factors into composite scales to assess for the risk of suicide following self-harm (Chan et al. 2016), and these are commonly used in clinical practice, with a wide variety of scales being used across different healthcare settings (Quinlivan et al. 2014). In a prison setting, this approach has been used for the identification of inmates that carried out suicide (Blaauw et al. 2005). A similar approach has also been utilised in order to identify self-harm (self-injurious behaviour) in male prisoners (Lanes 2009) (Barton et al. 2014). These studies produced AUC values of 0.89 (Lanes 2009) and 0.91 (Barton et al. 2014), with 93% (Lanes 2009) and 87% (Barton et al. 2014) of cases correctly classified, both of which are superior to the values obtained in the present study. However, both of these studies used retrospective data to classify the difference between prisoners with and without a history of self-harm, whereas the current study used prospective data to classify whether self-harm occurred among an ACCT population during an active follow-up period.

An alternative option to assessing the predictive capacity of available data would be to utilise a machine learning approach, where it is possible to discover relevant structural and/or temporal patterns in complex data which are often hidden and inaccessible to the human expert (Holzinger 2016). Machine learning approaches can often outperform conventional statistical predictive modelling in predicting health outcomes (Song et al. 2004), although this is often at the expense of being able to derive an exclamatory, interpretable model (Tiffin and Paton 2018). Should a machine learning approach be adopted, it would be recommended that a human aspect should remain in any final decision-making process.

Some of the predictive items identified within the present study differ from those that have previously been reported as risk factors for self-harm. For example, one study focusing on female incarcerated adults reported shame, anger and child abuse as important (Milligan and Andrews 2005). Although child abuse was not addressed, shame was incorporated as a question in our study, but it did not appear to be predictive of future self-harm. Additionally a ‘cry of pain’ model (i.e. trauma of first weeks of imprisonment) has been presented as a predictor of early self-harm in a male prison population (Slade et al. 2012). This was successful at predicting self-harm (with a rate of 97.7%) but used eight separate questionnaires, which may be unfeasible for routine use in most prison settings where both the prison regime and high turnover of prisoners leads to significant time constraints. A further study identified several independent predictors for suicide including previous psychiatric service contact, history of self-harm, single cell occupation, remand status, and non-white ethnicity (Humber et al. 2013). In the present study, history of self-harm was predictive, but remand status and non-white ethnicity were not predictive of self-harm. Previous contact with a psychiatrist was predictive for males and females, but cell occupancy status was not determined.

Some of the items identified in the present study are particularly interesting. For example, the finding in the male sample that alcohol abuse works in a ‘protective’ manner is contrary to the existing evidence base in mainstream populations, where problematic alcohol use is recognised as a risk factor for self-harm (Ness et al. 2015). Although there are various possible explanations for these findings, it is recommended that these items are studied further within this setting.

An issue with all risk factor item sets that have been derived in this way, as is the case in the present study, is that although these item sets seem to work statistically, it is likely that the identified items involve an element of capitalisation on chance within the specific dataset that is used. Due to this restriction, it is vital that any of these risk factor items sets are revalidated prospectively. Another major issue with a lot of the scales that have been derived in this way are that they use solely retrospective data, and they are never further validated prospectively, meaning that along with the chance capitalisation, no process of causality can be assumed.

Additionally, the practical implementation of risk factor item sets may be limited for a number of reasons. The identified risk factors are often comparatively common in the populations of interest (Chan et al. 2016), meaning that an impractical amount of false negatives would be identified. Another issue with the item set identified in the present study is that many of the items are static in nature. These static items refer to background and lifetime information which cannot change once the item has been affirmed. For example, for the item ‘Have you ever cut yourself on purpose?’, then if this has been affirmed then this response is fixed as it cannot be ‘undone’. This impracticality has been previously highlighted (Völlm and Dolan 2009), where it has been identified that although these simple check lists may be useful to identify those at risk of self-harm upon prison reception, this risk is not static; therefore risk assessment has to be a continuous process and should not be restricted to reception screening.

If an actual incidence of self-harm has occurred in order to trigger initiation of the ACCT, it has been suggested that a comprehensive psychosocial assessment of the risks and needs that are specific to the individual should be central to the management of these people who have self-harmed (Chan et al. 2016). This may be a plausible approach following a self-harm event, or perhaps if a prisoner had been identified as being at high risk of self-harm, but considering the limited resources within the prison system, the use of comprehensive assessment instruments would not be feasible in day-to-day practise, especially when being used for early risk assessment at prison reception (Völlm and Dolan 2009).

The gender-specific predictive risk item sets identified in this study may be useful in this regard, as they offer the opportunity to classify three levels differing levels of risk that could be used at reception into prison. If the risk classification was medium or high, then a further in-depth assessment could be carried out, as has been previously recommended (Chan et al. 2016). Given the high negative predictive values, the predictive item sets appear to function better at screening out self-harm than screening it in. This could therefore be potentially useful to assist the ‘sign-off’ from an ACCT, if the clinician or ACCT team worker deemed it safe to do so. Although this is not the ideal intention, it could still help to save time and focus the limited resources that are available.

Despite an apparently limited predictive power, the implementation of a screening process that is specific to self-harm could certainly contribute to an increased awareness of self-harm and mental health issues amongst prison staff. It has been identified that 29% of prison staff have not received any ACCT training, and 82% have not received any training in mental health awareness (Ward and Bailey 2013). This is consistent with other reports of a lack of staff training and policy, along with an inconsistency in response to self-harm behaviour (Roe-Sepowitz 2006). Additionally, in over 20% of suicide cases, non-medical staff had documented signs of suicidality, but no referral or further action was taken (Fruehwald et al. 2003). This evidence leads to the critical point that an improvement in staff awareness and attitude, along with further training, are important factors which may help prevent self-harm and suicide in prisons (Hawton et al. 2014; Humber et al. 2011; Saunders et al. 2012). Although this staff awareness shortfall has been identified and is being addressed, it has been acknowledged that much work remains to be done (Forrester and Slade 2014).

## Conclusions

Of the individuals starting on the ACCT process, almost 30% will go on to self-harm within six-months. None of the summary scores derived from the selected instruments showed a meaningful ability to predict self-harm, however, exploratory logistic regression analysis of individual background and instrument items revealed gender-specific item sets which were statistically significant in predicting future self-harm. However, as this analysis was carried out post-hoc, although it is plausible that these item sets could potentially be useful, their direct predictive capacity and operational functionality remains unknown.

## Abbreviations

ACCT: Assessment, Care in Custody, and Teamwork
AUC: Area under the curve
BHS: Beck Hopelessness Scale
BSL-23: Borderline Symptom List-23
BSL-23-F: Revised Borderline Symptom List-23 (frequency-based responses)
CORE-OM: Clinical Outcomes in Routine Evaluation System – Outcome Measure
DASS-21: Depression, Anxiety and Stress Scales-21
NPV: Negative-predictive value
PHQ-9: Patient Health Questionnaire-9
PPV: Positive-predictive value
PriSnQuest: Prison Screening Questionnaire
ROC: Receiver operating characteristic
SCOPE: Suicide Concerns for Offenders in Prison Environment
SH: Self-harm
SHI: Self-Harm Inventory
